# Supplementary material for: Structure of PDE3A-SLFN12 complex reveals requirements for activation of SLFN12 RNase
Source: Nat Commun. 2021 Jul 16;12:4375. doi: 10.1038/s41467-021-24495-w (PMC8285493; doi:10.1038/s41467-021-24495-w)
Supplement: Supplementary file 1 — Supplementary Information [file 41467_2021_24495_MOESM1_ESM.pdf]

Supplementary Figure 1. Analysis of the binding of DNMDP to PDE3A^{CAT} and SLFN12 and superposition of PDE3A$^{CAT\text{-}Xtl}$ structures.

a, SEC-MALS analysis of the PDE3A^{CAT}-SLFN12 complex. SEC traces for PDE3A^{CAT} (red), SLFN12 (green), and the complex (blue) are shown. The data for PDE3A and PDE3A+SLFN12 were collected at 150mM NaCl, and at 500 mM NaCl for SLFN12. The theoretical and experimentally determined mass of the proteins is shown in the table. **b,** DSC analysis of 4 μM PDE3A^{CAT} or **c,** SLFN12 in the absence (black line) and presence (gray line) of 10 μM DNMDP. The ΔT_m discussed in the manuscript was calculated based on the difference between the major peak of the traces for PDE3A^{CAT} ± DNMDP. It is not clear why we observe two peaks in the DSC. **(d,e)** Stereoviews of the superposition of the catalytic domain of PDE3A in the absence (blue), and presence of AMP (red), DNMDP (green), and trequinsin (cyan). The backbone of protein is shown in a ribbon representation and the AMP, DNMDP, and trequinsin in a licorice representation. The superposition of the whole chain is shown in **d** and an expanded version of the catalytic site in **e**.

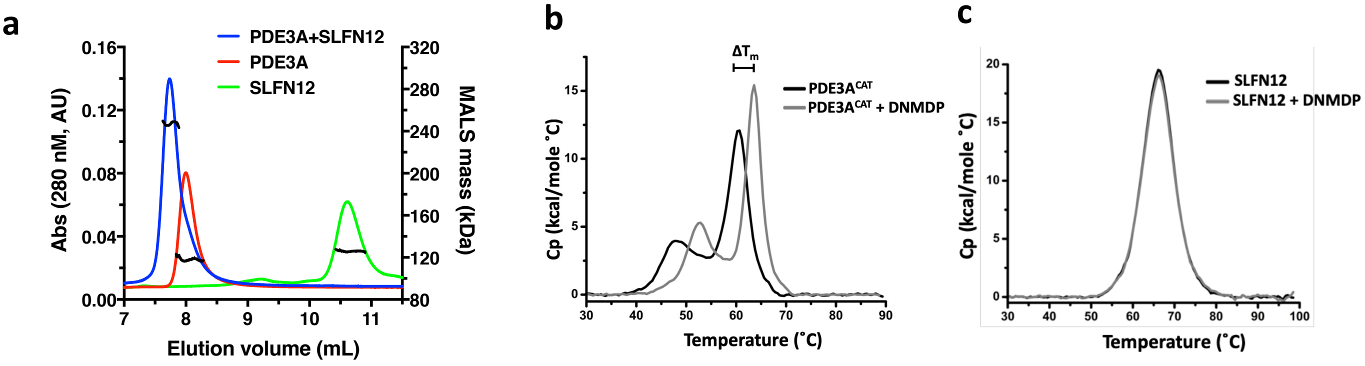

Protein	Theoretical Mass	SEC-MALS mass
$PDE3A_{640-1141}$	57.3 kDa (monomer)	118.0 kDa
SLFN12	67.3 kDa (monomer)	126.9 kDa
$PDE3A_{640-1141}$ + SLFN12	124.6 kDa (1:1 ratio)	246.9 kDa

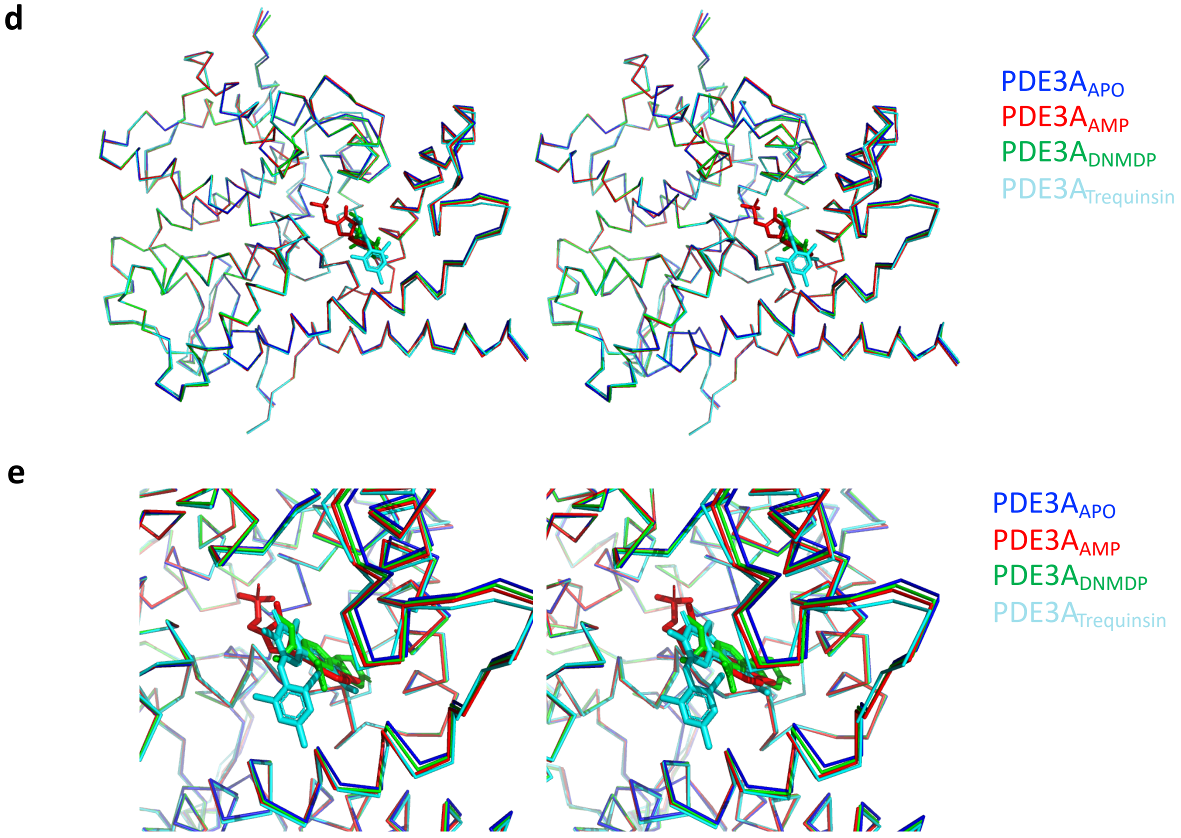

Supplementary Figure 1

Supplementary Figure 2. Time-resolved D-uptake heat maps of PDE3A^{CAT} alone and in the presence of SLFN12.

a, Heat map of the D-uptake observed for PDE3A^{CAT} (22 µM) bound to BRD9500 (40 µM). Prior to initiating deuterium exchange experiments, PDE3A^{CAT} and BRD9500 were incubated for 30 min at room temperature to ensure complex formation. Color-coding is based on the deuterium uptake across different time points, with slow-exchanging regions represented with blue and fast exchanging regions with red. **b,** Peptide map and differential D-uptake of PDE3A^{CAT}:BRD9500 in the presence of SLFN12. Proteins were mixed in equimolar concentrations and incubated for 30 min on ice to ensure complex formation. Out of the 750 peptides identified (horizontal lines), 452 peptides were analyzed for their D-content, yielding an almost complete sequence coverage and an average redundancy value of ~14. Peptides for which a % deuterium uptake difference was observed between the two protein states are depicted with green and blue. Peptides in grey indicate that their D-uptake was unaffected in the presence of SLFN12. The heat map was generated in HDExaminer as follows: First, the protein was divided into non-overlapping “atomic peptides”. These atomic peptides were formed by dividing the protein everywhere an observed peptide starts or ends. Each observed peptide’s deuteration level was then expressed as a sum of the deuteration levels for one or more atomic peptides. The first two residues of each peptide were ignored, since these are widely considered to exchange too rapidly. The deuteration level for each atomic peptide was subsequently computed by minimizing the least squares error within the set of observed peptides. For mapping onto the heat map and the PDE3A structure, deuteration levels were further smoothed in HDExaminer. **c,** Residual plot depicting D-uptake differences between PDE3A^{CAT}-BRD9500 and PDE3A^{CAT}-BRD9500-SLFN12. Each dot represents a unique peptide shown for different time points. Negative values indicate a decrease in the D-uptake of PDE3A^{CAT} in the presence of SLFN12 and could suggest a probable interaction interface. Deuterium differences larger than 4% are considered significant.

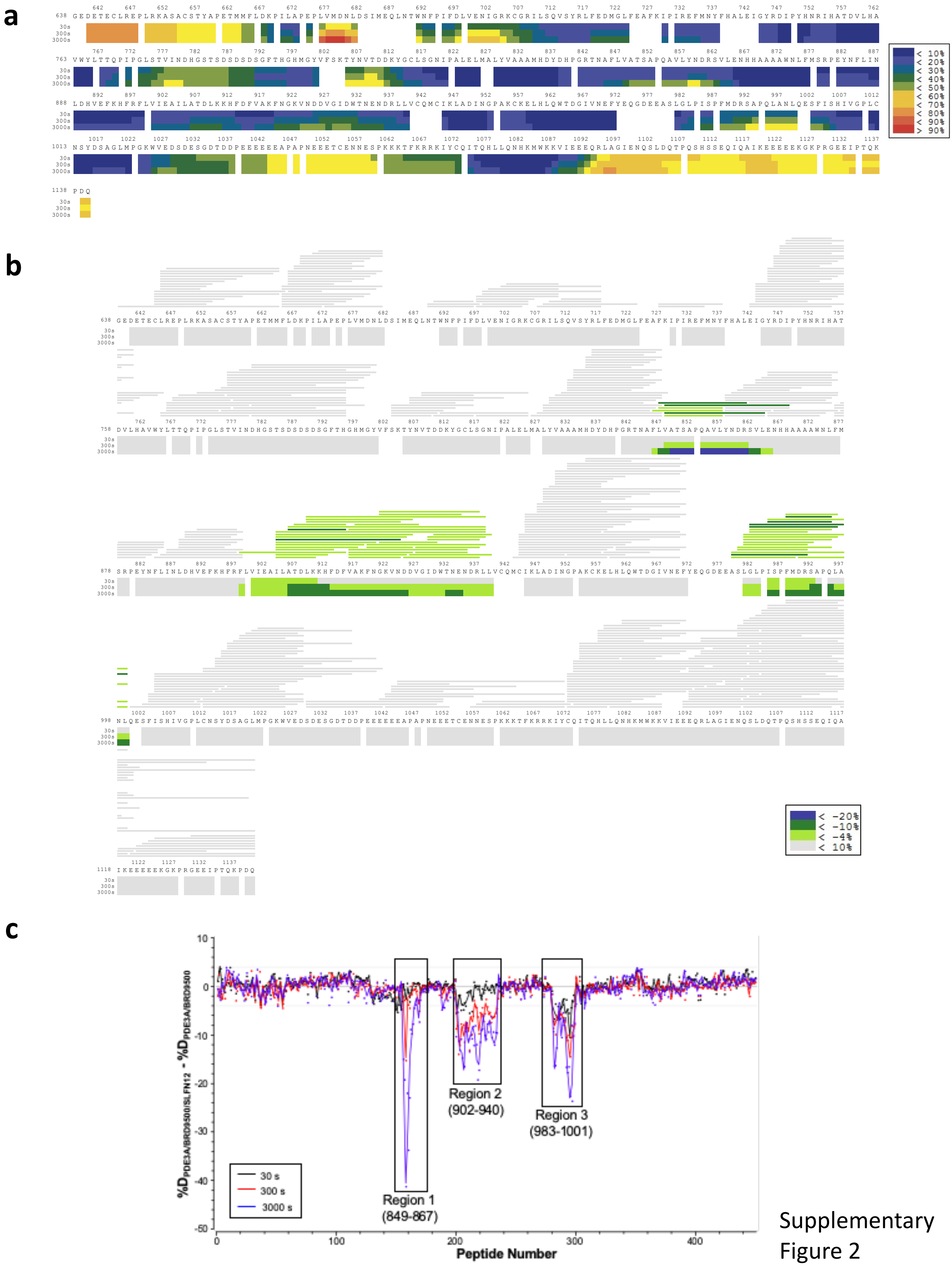

Supplementary Figure 2

Supplementary Figure 3. Cryo-EM data processing workflow.

Following generation of an *ab initio* initial 3D reference model, 3D classification with global particle pose searches was used to filter remaining junk particles. This particle set was improved by refining per-particle defocus and per-optics group beamtilt parameters. These were then used as the input to the Relion "Bayesian polishing" per-particle motion correction procedure. 3D classification (with fixed particle poses) of the polished particles was then used to eliminate outlier conformations of the complex. Selected polished particles were then improved over three rounds of refinement of all applicable CTF parameters. Remaining conformational heterogeneity was resolved by Relion multi-body refinement with SLFN12 and PDE3A as two separate bodies. A particle subset representing particles where both bodies were positioned along a common central symmetry axis was used for a final 3D refinement. The imposed symmetry and resulting resolution (at half-maps FSC=0.143) are noted for each refinement. All steps were performed in the presence of a solvent mask unless noted. The masks used to delineate the SLFN12 and PDE3A bodies for multi-body refinement are shown as color-coded meshes to the left of the resulting refined bodies.

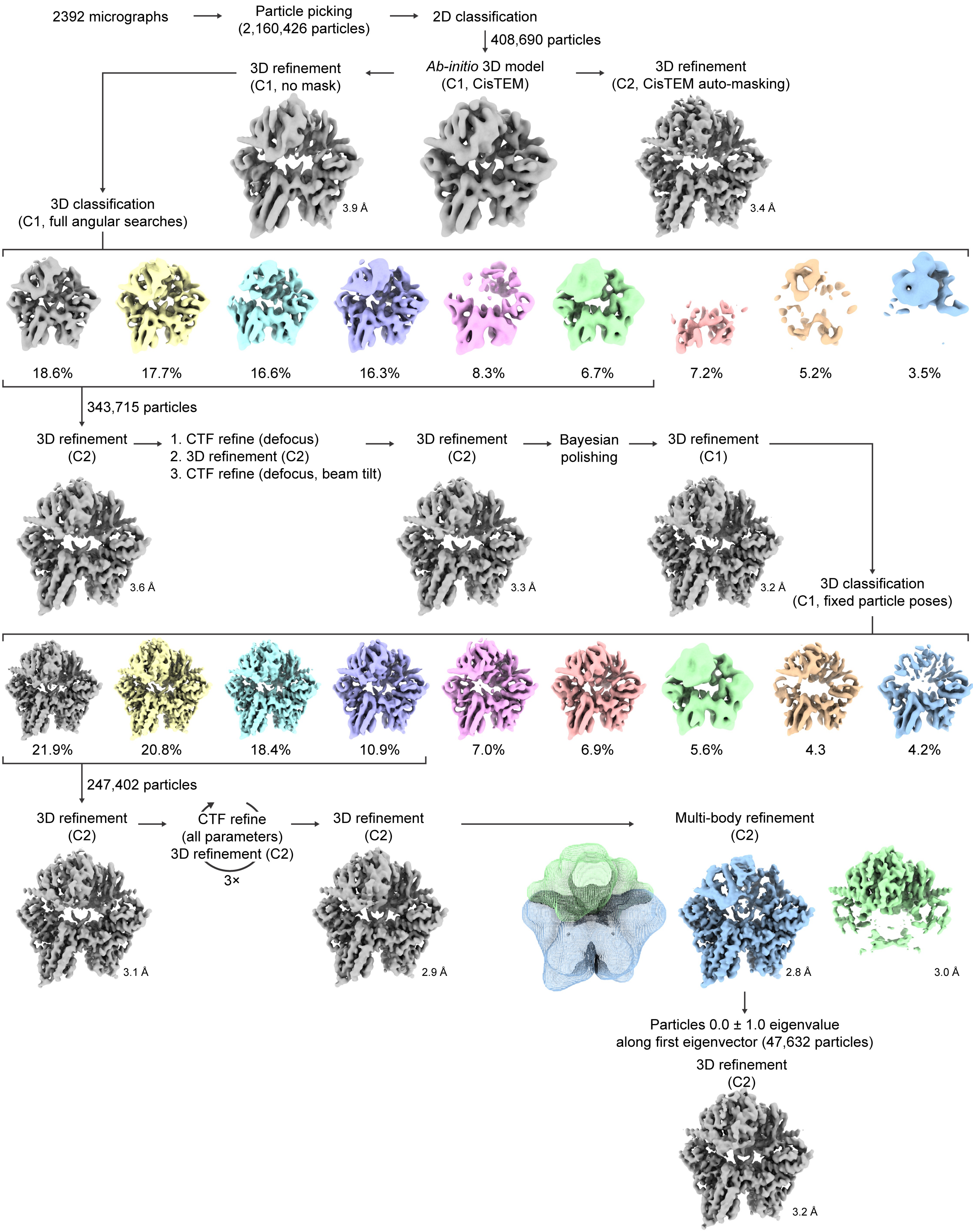

Supplementary Figure 3

Supplementary Figure 4. Resolution estimates and angular distribution for the PDE3A^{CAT}-SLFN12 cryo-EM maps.

Three maps were used for construction of the PDE3A^{CAT}-SLFN12 model: one for each protein dimer body, which were used for atomic model building and refinement, and a consensus subset of particles in which the two bodies share the same twofold symmetry axis, which was used to merge the two dimer models. **a,** Maps colored by local resolution, as calculated by the method implemented in RELION. **b,** Gold-standard half-map Fourier shell correlation (FSC) (blue) and map-model FSC (orange) curves for each map and model. Resolutions are given at the standard cutoffs (FSC = 0.143 and 0.5, respectively). **c,** The angular particle distribution in the consensus subset map, shown in two 90°-separated views. **d,** Representative experimental cryo-EM density centered around Tyr830 of the hydrophobic core of PDE3A (blue sticks); **e,** Trp428 of the NTD of SLFN12 that was built de novo (green sticks); and **f,** DNMDP (cyan sticks). All maps were sharpened by an automatically determined B-factor and filtered to local resolution.

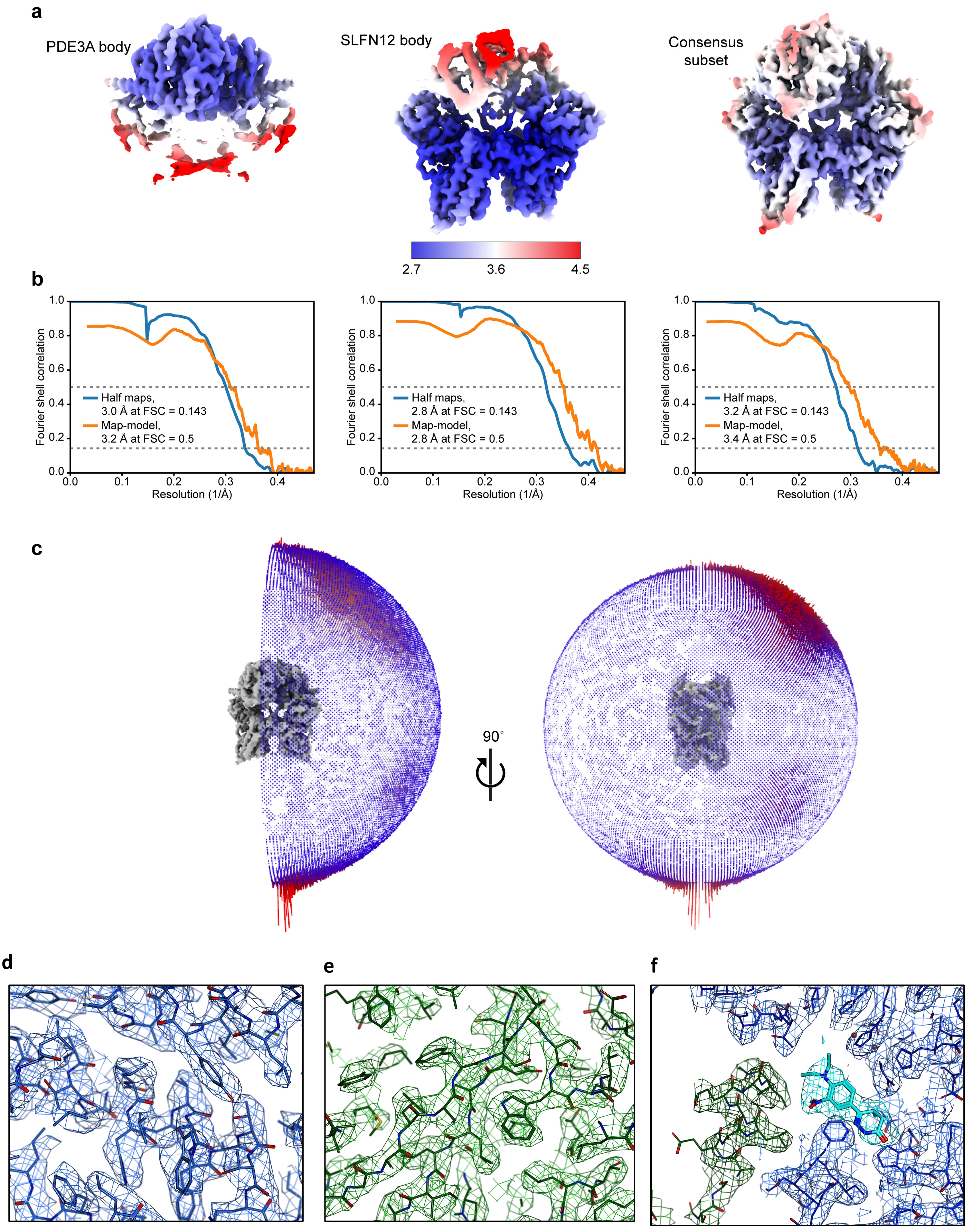

Supplementary Figure 4

Supplementary Figure 5. Further analysis of the PDE3A and SLFN12 structure.

a, Stereoview of the superposition of the catalytic domains of PDE3A from the apo crystal structure (green) and cryo-EM structure (red). **b,** Tertiary and secondary structure of the N-terminal and C-terminal domains of the SLFN12 monomer. The tertiary fold of the regions of the N-terminal domain and the C-terminal domain are shown at the top with the topology diagrams below. The a-helices are colored red and the b-strands are colored yellow. In the topology diagram, the a-helices and b-strands are represented as rectangles and arrows, respectively. The a-helices and b-strands are numbered from N-terminus to C-terminus. The zinc ion is shown as a gray sphere with the four coordinating residues indicated. **c,** Comparison of the N-terminal domains from the cryo-EM SLFN12 and crystal structure of SLFN13. The structures of the N-terminal domains (NTD) from the cryo-EM SLFN12 and crystal structure of SLFN13 are shown on the left and right, respectively. The structures are shown as cartoon and (transparent) surface representations. The regions of the NTD are colored as shown in Figure 5. For SLFN12, the width of the cavity between the N-lobe and C-lobe was measured using the Ca atom from Lys 35 and Lys 213. For SLFN13, Lys 36 and Arg 218 were used. **d**, Location of residues E200 and E205 of SLFN12 relative to PDE3A. SLFN12 is shown semi-transparent to highlight the position of E200, and E205, which are shown in a van der Waals surface representation. **e,** Conformation of the linker between the core domain of SLFN12-CTD and the SLFN12-PIR. The interactions are centered around Phe 548. **f,** Interactions between the SLFN12-NTD and SLFN12-CTD. The interactions are centered around two residues: Ala 12 (left) and Pro 519 (right). The residues are colored red or cyan depending on whether they are derived from SLFN12-NTD or SLFN12-CTD, respectively.

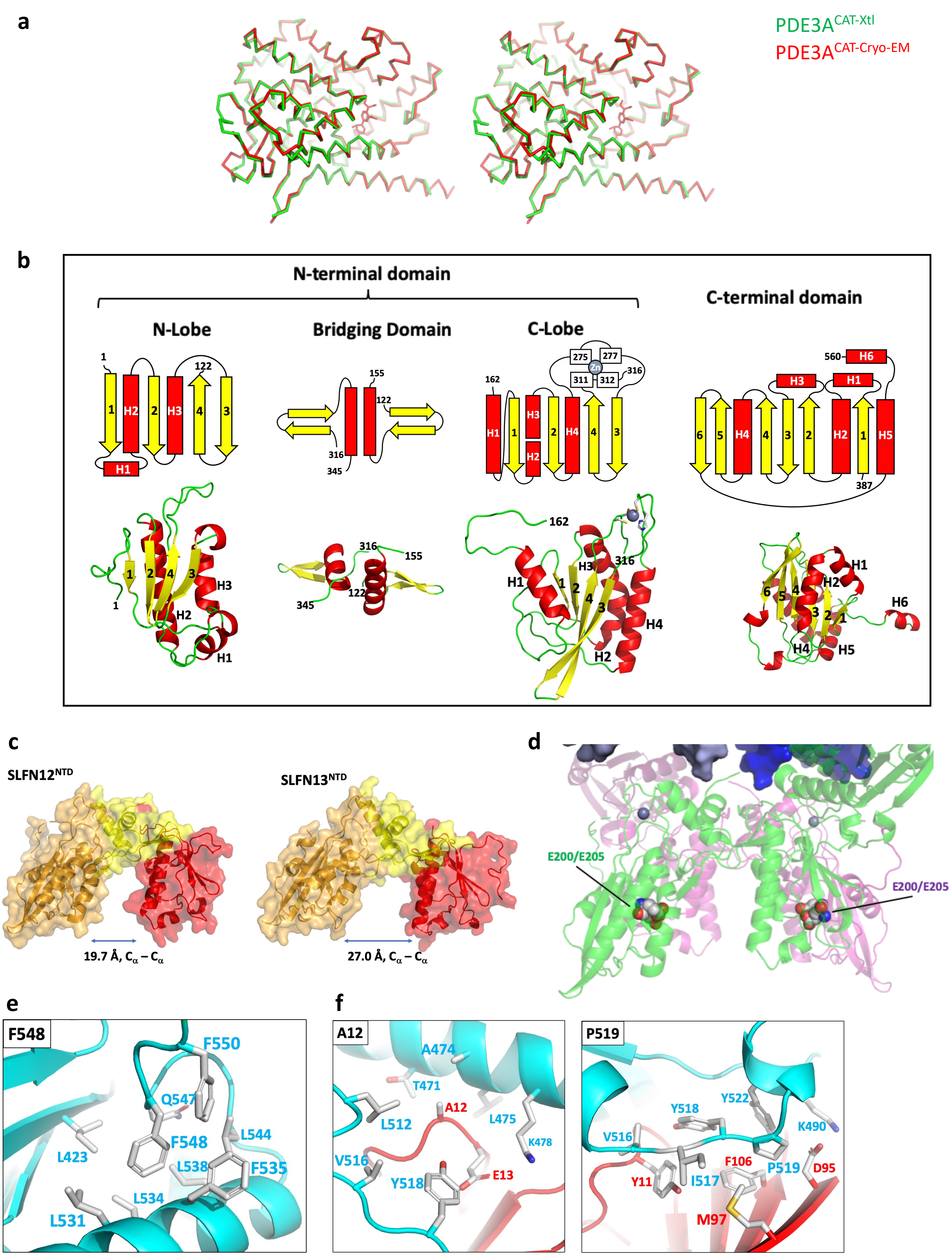

Supplementary Figure 5

Supplementary Figure 6. CRISPR knockout of PDE3A in GB1 cells for deep mutational scanning assay.

a, Immunoblot of PDE3A in parental GB1 cells and three clonal PDE3A CRISPR knockout GB1 cells lines. This immunoblot was performed once, but lack of PDE3A expression in sg6F12 relative to parental GB1 cells was confirmed in a second biological replicate and immunoblot. **b,** 72-hour DNMDP dose response of parental and PDE3A-KO GB1 cell lines, with or without ectopic expression of PDE3A transduced at a multiplicity of infection of 1. **c,** Sequencing of genomic PDE3A from CRISPR-KO PDE3A cell line sg6F12 showing frequencies of specific editing events of the four alleles of PDE3A in GB1 cells. **d,** Change in PDE3A allele abundance under negative control conditions. No change is expected; variable sites indicate regions of low coverage in the mutant allele library subsequently eliminated from analysis.

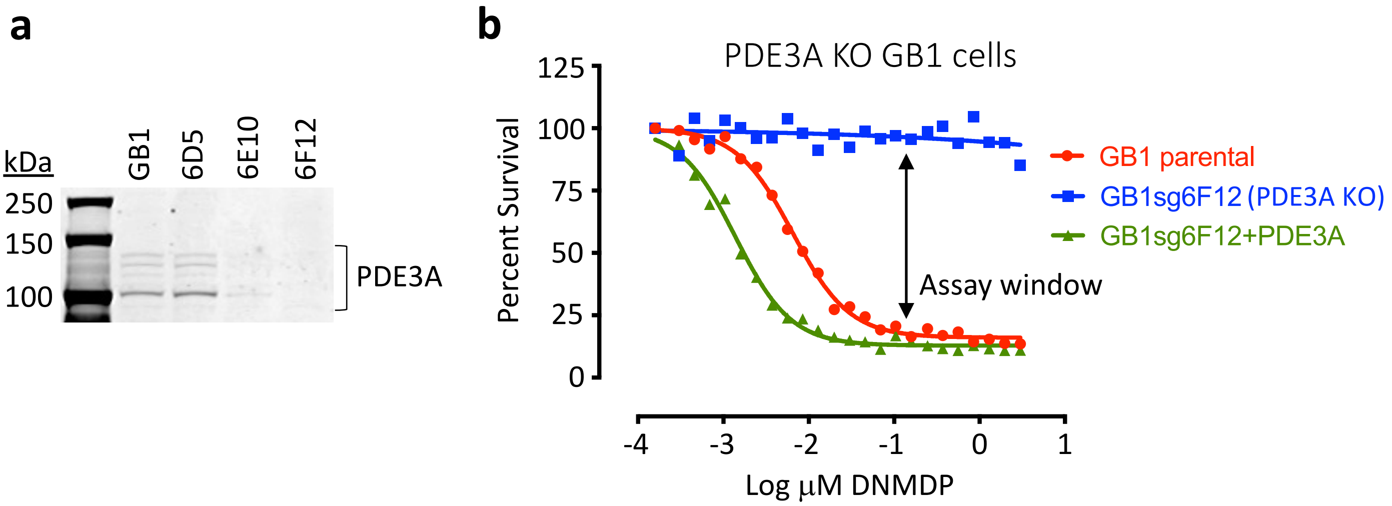

c

GB1sg6F12

```
Guide RNA                                GTGGCAGACCATATTCCCAAGGG
PDE3A ref    TTCCAATATTTTTTAGCTGTGGCAGACCATATTCCCAAGGGAATCCTGCTGATGAGCCCCTGGA
54% edited   TTCCAATATTTTTTAGCTGTGGCAGACCATATTCC-AAGGGAATCCTGCTGATGAGCCCCtgga
25% edited   TTCCAATATTTTTTAGCTGTGGCAGACCA---TCC-AAGGGAATCCTGCTGATGAGCCCCtgga
21% edited   TTCCAATATTTTTTAGCTGTGGCAGACCATATT-----------CCTGCTGATGAGCCCCtgga
Pam mutant                                                    GGN
```

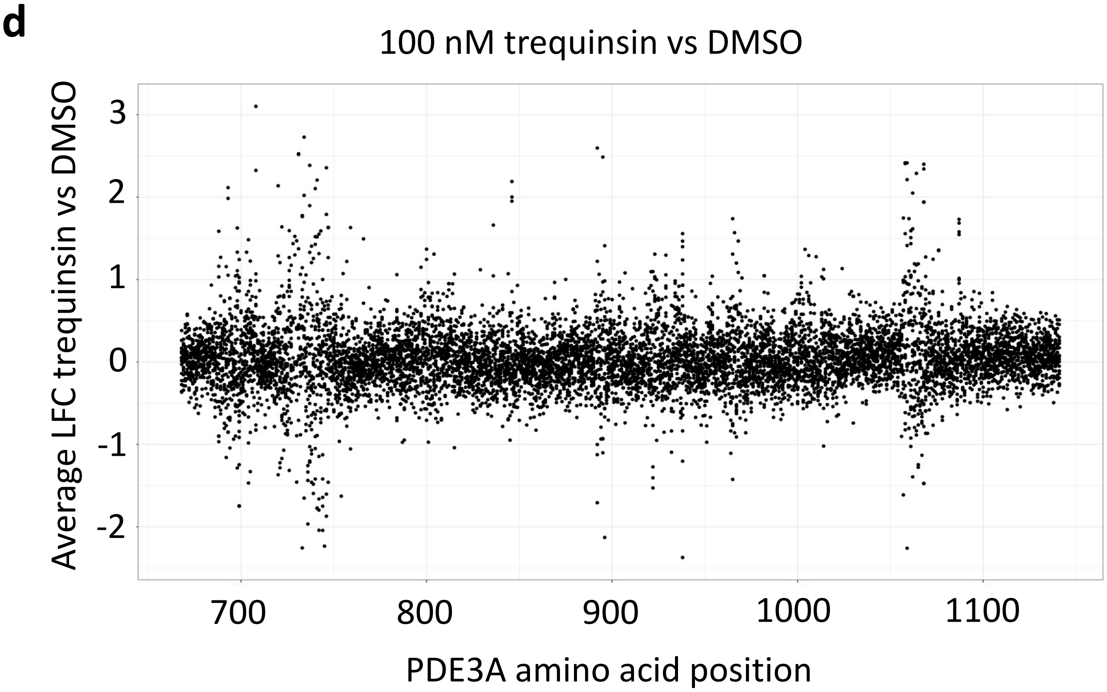

Supplementary Figure 6

Supplementary Figure 7. Multiple sequence alignment between the RNase regions of rat SLFN13, human SLFN13, and human SLFN12.

SLFN13 catalytic residues are marked in red, conserved charged residues in green, non-conserved charged residues in blue, and the CCCH zinc finger is marked by blue circles.

```
                                                   K38 R39 K42
rat SLFN13    -MEIHPSLVVEPSYPDLIIHAGEVTLGEKDRNKMDSKKK-RLEKARITEAACALLNSGGG 58
human SLFN13  MEANHCSLGVYPSYPDLVIDVGEVTLGEENRKKLQKTQR-DQERARVIRAACALLNSGGG 59
human SLFN12  ---MNISVDLETNYAELVLDVGRVTLGENSRKKMKDCKLRKKQNESVSRAMCALLNSGGG 57
                  : *: :  .* :*::..*.*****:.*:*:.. :    :.  : .* *********

rat SLFN13    VIVMQMSNKSEHPV--EMGLDLETSLRELIPSSDLQAFIETKQQGDLFYIFVKSWSCSP- 115
human SLFN13  VIQMEMANRDERPT--EMGLDLEESLRKLIQYPYLQAFFETKQHGRCFYIFVKSWSGDPF 117
human SLFN12  VIKAEIENEDYSYTKDGIGLDLENSFSNILL--FVPEYLDFMQNGNYFLIFVKSWSLNT- 114
              **  :: *..   .   :***** *: :::    :  :::  *:*  * ******* .

rat SLFN13    -KDGSTKPRICSLSSSLYCRSLTSKLPLDSKETFEFLERKKTCVKGSLTDGKGPPAKIPR 174
human SLFN13  LKDGSFNSRICSLSSSLYCRSGTSVLHMNSRQAFDFLKTKERQSKYNLINEGSPPSKIMK 177
human SLFN12  -----SGLRITTLSSNLYKRDITSAKVMNATAALEFLKDMKKTRGR-LYLRPELLAKRPC 168
                      ** :***.** *. **   :::  :::**:  :      *       :*
                                         E205 E210      R217   K224
rat SLFN13    LMYQNDL-ESNPAFEIFQSERLEYGQRLPFSESASIEFKQFSTRRAHEYIKSVIPEYISA 233
human SLFN13  AVYQNIS-ESNPAYEVFQTDTIEYGEILSFPESPSIEFKQFSTKHIQQYVENIIPEYISA 236
human SLFN12  VDIQEENNMKALAGVFFDRTELDRKEKLTFTESTHVEIKNFSTEKLLQRIKEILPQYVSA 228
                 *:    .  *  .*:   ::  : * * **  :*:*:***.:  : ::.::*:*:**
                             D248                           K276
rat SLFN13    FANTQGGYLLFGVDDESKRVLGCPKDNVDRDSLKAVVNEAISKLPVFHFCSSKEKVSYKT 293
human SLFN13  FANTEGGYLFIGVDDKSRKVLGCAKEQVDPDSLKNVIARAISKLPIVHFCSSKPRVEYST 296
human SLFN12  FANTDGGYLFIGLNED-KEIIGFKAEMSDLDDLEREIEKSIRKMPVHHFCMEKKKINYSC 287
              ****:****::*:::. :.::*   :  * *.*:  :  .:* *:*: *** .* ::.*.

rat SLFN13    RVIDVFKEGNLYGYLCVIKVERFCCAVFSEAPISWMADKENGVYSLNTEKWVRMMVDIGP 353
human SLFN13  KIVEVFCGKELYGYLCVIKVKAFCCVVFSEAPKSWMVRE-KYIRPLTTEEWVEKMMDADP 355
human SLFN12  KFLGVYDKGSLCGYVCALRVERFCCAVFAKEPDSWHVKD-NRVMQLTRKEWIQFMVEAEP 346
              :.: *:   .* **:*.::*: ***.**:: * ** . . : :  *. ::*:. *::  *
```

Supplementary Figure 7

Trequinsin IC50s (N=1)

Phosphodiesterase	IC50 (M)	Species
PDE1	8.80E-06	Bovine
PDE2A	7.85E-07	Human
PDE3A	2.30E-11	Human
PDE3B	5.00E-11	Human
PDE4B	3.20E-07	Human
PDE5	8.70E-07	Human
PDE6	1.20E-06	Bovine
PDE7B	>1.00E-5	Human
PDE8A	8.00E-06	Human
PDE9A	>1.00E-5	Human
PDE10A	4.98E-08	Human
PDE11A	1.00E-06	Human

Supplementary Table 1. IC_{50} data for trequinsin across phosphodiesterases.

Data Collection*				
	**PDE3A(APO)**	**PDE3A+DNMDP**	**PDE3A+cAMP**	**PDE3A+Trequinsin**
**Space group**	$P2_1$	$P2_1$	$P2_1$	$P2_1$
**Cell dimensions (Å)**	82.37, 58.79, 157.66	82.21, 58.52, 157.54	82.42, 58.78, 157.0	83.24, 59.65, 158.12
**Unit cell angles (˚)**	90, 90.69, 90	90, 90.66, 90	90, 90.74, 90	90, 90.48, 90
**Wavelength (Å)**	0.976	0.976	0.976	0.976
**Resolution (Å)**	47.13 - 1.702 (1.763 - 1.702)	47.67 - 2.002 (2.074 - 2.002)	47.86 - 2.08 (2.154 - 2.08)	41.24 - 2.204 (2.283 - 2.204)
**R_{meas} (%)**	8.052 (122.5)	6.753 (93.7)	10.3 (127.3)	6.562 (67.42)
**I/σ (I)**	8.69 (0.92)	11.48 (1.51)	9.74 (1.25)	11.29 (1.74)
**Completeness (%)**	99.15 (97.96)	94.3 (91.7)	99.50 (98.94)	98.46 (96.97)
**Refinement**				
**Resolution (Å)**	47.13 - 1.702 (1.763 - 1.702)	47.67 - 2.002 (2.074 - 2.002)	47.86 - 2.08 (2.154 - 2.08)	41.24 - 2.204 (2.283 - 2.204)
**No. reflections**	164233 (16112)	100572 (9898)	90404 (8928)	77687 (7573)
**R_{work} (%)**	20.06 (38.11)	19.26 (31.79)	23.08 (36.48)	23.11 (38.85)
**R_{free} (%)**	22.35 (39.58)	22.20 (35.35)	24.77 (37.45)	25.54 (39.55)
**Average B-Factor (Å^2)**	36.00	46.20	46.04	60.82
**Bond lengths (Å)**	0.011	0.016	0.012	0.010
**Bond angles (˚)**	1.32	1.94	1.51	1.34
**Ramachandran**				
**Favored (%)**	97.84	97.22	97.31	94.69
**Allowed (%)**	1.81	2.16	2.41	3.03
**Outliers (%)**	0.35	0.63	0.28	0.48

Supplementary Table 2. Crystallographic statistics for data processing and refinement.

	SLFN12 body	PDE3A body	Composite model
**Map resolution (FSC = 0.143) (Å)**	2.76	2.97	3.22
**Map symmetry applied**	C2	C2	C2
**Map-model fit (unsharpened/sharpened-filtered**			
**FSC = 0.5 (Å)**	2.8 / 2.8	3.2 / 3.0	3.4 / 3.3
**Masked cross correlation**	0.83 / 0.85	0.75 / 0.81	0.76 / 0.78
**Composition (#)**			
**Chains**	2	4	4
**Atoms (no hydrogens)**	8142	6202	14344
**Protein residues**	1004	762	1766
**Ligands**	Zn: 2	DNMDP: 2<br>Mn: 2<br>Mg: 2	Zn: 2<br>DNMDP: 2<br>Mn: 2<br>Mg: 2
**Bonds (RMSD)**			
**Length (Å) (no. > 4 σ)**	0.002 (0)	0.002 (0)	0.002 (0)
**Angles (˚) (no. > 4 σ)**	0.422 (0)	0.408 (0)	0.425 (0)
**Dihedral Angles (˚) (no. > 4 σ)**	7.411 (0)	7.782 (0)	7.639 (0)
**MolProbity score**	1.14	1.29	1.23
**Clash score**	3.54	5.32	4.58
**EMRinger score**	4.38	3.03	2.87
**Ramchandran plot (%)**			
**Outliers**	0.00	0.00	0.00
**Allowed**	1.61	0.80	1.27
**Favored**	98.39	99.20	98.73
**Rotamer outliers (%) (no.)**	0.22 (2)	0.30 (2)	0.25 (4)
**Cß outliers (%) (no.)**	0.00 (0)	0.00 (0)	0.00 (0)
**Peptide plane (%)**			
**Cis proline/general**	10.0 / 0.0	0.0 / 0.0	3.6 / 0.0
**Twisted proline/general**	0.0 / 0.0	0.0 / 0.0	0.0 / 0.0
**ADP (B-factors) vs sharpened-filtered map (min/max/mean)**			
**Protein**	20.85/97.43/49.84	22.50/96.99/45.08	20.85/97.43/47.79
**Ligand**	57.67/58.34/58.01	29.14/48.86/39.63	29.14/58.34/40.37

Supplementary Table 3. CryoEM statistics for refinement of complex structure.

Residue no.	Amino acid	Mutation introduced	PDE3A homodimerization	Interaction with DNMDP	SLFN12 interacting residue
857	V	P, D	Yes	No	No
858	L	E, D, P, G	Yes	No	No
859	Y	R, L, W, K, N, G, S, P, T, C, E, I, V, Q, D, M	Yes	No	No
860	N	M, D, I, R, K, V, W, Y, G, E, F, Q, T, L, A, P	Yes	No	No
861	D	I, V, A, K, P, S, R, T	Yes	No	No
867	N	W, I, K, G, L, V, T, D, P, R	Yes	No	No
871	A	L, E, I, P, D, V, M	Yes	No	No
910	L	R, K, G, S, E, N, Q, D	No	Yes	I558
911	K	D, E, P	No	No	I558
914	F	C, E, A, Q, S, T, N, R, K, G, D, V	No	No	Y555 and I560
972	F	Y, H, K, P, Q, E, D, R	No	Yes	I557
988	P	E, D, L, K, R, Q	No	No	N553, A556, and I557
989	F	N, R, G, E, K, P, Q, I, D	No	No	N553, L554, and I557
990	M	D, N, Q, E, Y, W, P, V, R, K, T	No	No	I557
1000	L	Y, T, S, R, P, E, D, A, K, G	No	No	L554
1003	S	W	No	No	A551
1004	F	N, R, K, M, E, G, D, Y, P, A, Q, H, Z, S, T, W	No	Yes	L554
1007	H	P	No	No	L554
1008	I	N, E, A, W, K, D, G, Y, H, R, T, Q, S, P	No	No	I558

Supplementary Table 4. Missense mutations of residues located in the PDE3A homodimerization and PDE3A-SLFN12 interfaces causing DNMDP resistance in the deep mutational scanning assay.

Primer Name	Primer Sequence	Comments
SLFN12_R43S_F	AAAGCAGAATGAAAGcGTCTCACGAGCTATG	Used with SLFN12_R168C_R, GeneArt PLUS mutagenesis PCR
SLFN12_R43S_R	CATAGCTCGTGAGACgCTTTCATTCTGCTTT	Used with pDONR_3500_F, GeneArt PLUS mutagenesis PCR
SLFN12_R168C_F	CTGGCAAAGAGGCCCtGTGTTGATATACAAG	Used with pDONR_3500_R, GeneArt PLUS mutagenesis PCR
SLFN12_R168C_R	CTTGTATATCAACACaGGGCCTCTTTGCCAG	Used with SLFN12_R43S_F, GeneArt PLUS mutagenesis PCR
SLFN12_E200A_F	GAAAAATTGACCTTTACTGcATCCACACATGTTGA	Used with pDONR_3500_R, GeneArt PLUS mutagenesis PCR
SLFN12_E200A_R	TCAACATGTGTGGATgCAGTAAAGGTCAATTTTTCT	Used with pDONR_3500_F, GeneArt PLUS mutagenesis PCR
SLFN12_E205A_F	GAATCCACACATGTTGcAATTAAAAACTTCTCGACAG	Used with pDONR_3500_R, GeneArt PLUS mutagenesis PCR
SLFN12_E205A_R	CTGTCGAGAAGTTTTTAATTgCAACATGTGTGGATTC	Used with pDONR_3500_F, GeneArt PLUS mutagenesis PCR
SLFN12_E200A_E205A_F	GAAAAATTGACCTTTACTGcATCCACACATGTTGcAATTAAAAACTTCTCGACAG	Used with pDONR_3500_R, GeneArt PLUS mutagenesis PCR
SLFN12_E200A_E205A_R	CTGTCGAGAAGTTTTTAATTgCAACATGTGTGGATgCAGTAAAGGTCAATTTTTCTT	Used with pDONR_3500_F, GeneArt PLUS mutagenesis PCR
pDONR_3500_F	gaggcgctaaatgaaaccttaacgctatgg	Used with SLFN12 mutagenic Reverse primers, GeneArt PLUS mutagenesis PCR
pDONR_3500_R	ccatagcgttaaggtttcatttagcgcctc	Used with SLFN12 mutagenic Forward primers, GeneArt PLUS mutagenesis PCR
PDE3A_F914D_F	GAAGAAACACgatGACTTCGTAGCC	Used with PDE3A_GW_R2, overlapping PCR mutagenesis and Gateway BP reaction
PDE3A_F914D_R	GGCTACGAAGTCatcGTGTTTCTT	Used with PDE3A_GW_F, overlapping PCR mutagenesis and Gateway BP reaction
PDE3A_F914A_F	GAAGAAACACgctGACTTCGTAGCC	Used with PDE3A_GW_R2, overlapping PCR mutagenesis and Gateway BP reaction
PDE3A_F914A_R	GGCTACGAAGTCagcGTGTTTCTT	Used with PDE3A_GW_F, overlapping PCR mutagenesis and Gateway BP reaction
PDE3A_N867R_F	TCAGTTTTGGAGagaCATCACGCAGCTGCT	Used with PDE3A_GW_R2, overlapping PCR mutagenesis and Gateway BP reaction
PDE3A_N867R_R	GCAGCAGCTGCGTGATGtctCTCCAAAACTGA	Used with PDE3A_GW_F, overlapping PCR mutagenesis and Gateway BP reaction
PDE3A_GW_F	GGGGACAACTTTGTACAAAAAAGTTGGCaccATGGCAGTGCCCGGCGACGCTGC	Used with PDE3A mutagenic Reverse primers, overlapping PCR mutagenesis and Gateway BP reaction
PDE3A_GW_R2	GGGGACAACTTTGTACAAGAAAGTTGGcaaCTGGTCTGGCTTTTGGGTTGG	Used with PDE3A mutagenic Forward primers, overlapping PCR mutagenesis and Gateway BP reaction
3XFLAG_EcoRV_AvrII_SpeI_F	ATCGACTACAAGGACCACGACGGTGACTACAAGGACCACGACATCGACTACAAGGACGACGACGACAAGTGATAATGACCTAGGA	Annealed with 3XFLAG_EcoRV_AvrII_SpeI_R, restriction cloning
3XFLAG_EcoRV_AvrII_SpeI_R	CTAGTCCTAGGTCATTATCACTTGTCGTCGTCGTCCTTGTAGTCGATGTCGTGGTCCTTGTAGTCACCGTCGTGGTCCTTGTAGTCGAT	Annealed with 3XFLAG_EcoRV_AvrII_SpeI_F, restriction cloning
AmpR_F	GGATGGAGGCGGATAAAGTTGCAGG	Used with TetOne_BBamplification_R, Gibson assembly
TetOne_BBamplification_R	GAATTCTTTACGAGGGTAGGAAGTG	Used with AmpR_F, Gibson assembly
TetOne_BBamplification_F	GGATCCAGACCACCTCCCCTGCGAG	Used with AmpR_R_2, Gibson assembly
AmpR_R_2	CCTGCAACTTTATCCGCCTCCATCCAG	Used with TetOne_BBamplification_F, Gibson assembly
TetOne_GW_Gib_F	CACTTCCTACCCTCGTAAAGAATTCacaagtttgtacaaaaaagctg	Used with TetOne_GW_Gib_R_FLAG, Gibson assembly
TetOne_GW_Gib_R_FLAG	CGCAGGGGAGGTGGTCTGGATCCttactaTCACTTGTCGTCGTCGTCCTTGTAGTC	Used with TetOne_GW_Gib_F, overlapping PCR mutagenesis and Gateway BP reaction
SLFN12_kozak_GW_F	GGGGACAACTTTGTACAAAAAAGTTGGCaccATGAACATCAGTGTTGATTTGGAAACG	Used with SLFN12 mutagenic Reverse primers, overlapping PCR mutagenesis and Gateway BP reaction
SLFN12_GW_Ro	GGGGACAACTTTGTACAAGAAAGTTGggcaGGTGAGCCTTCGAC	Used with SLFN12 mutagenic Forward primers, overlapping PCR mutagenesis and Gateway BP reaction
SLFN12_1-548_GW_Ro	GGGGACAACTTTGTACAAGAAAGTTGggcaAAACTGGTCTCTCAG	Used with SLFN12_kozak_GW_F, PCR and Gateway BP reaction
SLFN12_1-568_GW_Ro	GGGGACAACTTTGTACAAGAAAGTTGggcaTTTATCATTCTTCTG	Used with SLFN12_kozak_GW_F, PCR and Gateway BP reaction

Supplementary Table 5. DNA oligonucleotide sequences used in this study.

**Protein**	**Codon-optimized cDNA sequence**
PDE3A(640-1141)	GAAGATGAAACCGAATGTCTGCGTGAACCGCTGCGTAAAGCAAGCGCATGTAGCACCTATGCACCG<br>GAAACCATGATGTTTCTGGATAAACCGATTCTGGCACCGGAACCGCTGGTTATGGATAATCTGGAT<br>AGCATTATGGAACAGCTGAACACCTGGAACTTTCCGATTTTTGATCTGGTGGAAAACATTGGTCGT<br>AAATGTGGTCGTATTCTGAGCCAGGTTAGCTATCGTCTGTTTGAAGATATGGGTCTGTTCGAAGCC<br>TTTAAAATCCCGATTCGTGAATTTATGAACTACTTTCATGCCCTGGAAATTGGCTATCGTGATATT<br>CCGTATCATAATCGTATTCATGCAACCGATGTTCTGCATGCCGTTTGGTATCTGACCACCCAGCCG<br>ATTCCGGGTCTGAGCACCGTTATTAATGATCATGGTAGCACCAGCGATAGCGATTCAGATAGCGGT<br>TTTACCCATGGTCATATGGGTTATGTGTTCAGCAAAACCTATAACGTGACCGATGATAAATATGGT<br>TGTCTGAGCGGTAATATTCCGGCACTGGAACTGATGGCACTGTATGTTGCAGCAGCAATGCATGAT<br>TATGATCATCCGGGTCGTACCAATGCATTTCTGGTTGCGACCAGCGCACCGCAGGCAGTTCTGTAT<br>AATGATCGTAGCGTTCTGGAAAATCATCATGCAGCAGCCGCATGGAACCTGTTTATGAGCCGTCCG<br>GAATATAACTTTCTGATCAATCTGGATCATGTGGAATTCAAACACTTTCGCTTTCTGGTGATTGAA<br>GCAATTCTGGCAACCGATCTGAAAAAACACTTTGATTTTGTGGCCAAATTTAACGGCAAAGTGAAT<br>GATGATGTGGGCATTGATTGGACCAATGAAAATGATCGTCTGCTGGTTTGTCAGATGTGTATTAAA<br>CTGGCCGATATCAATGGTCCGGCAAAATGTAAAGAACTGCATCTGCAGTGGACCGATGGTATTGTG<br>AATGAATTTTATGAGCAGGGTGATGAAGAAGCAAGCCTGGGTCTGCCGATTAGCCCGTTTATGGAT<br>CGTAGTGCACCGCAGCTGGCAAATCTGCAAGAAAGCTTTATTAGCCATATTGTTGGTCCGCTGTGC<br>AATAGCTATGATAGCGCAGGTCTGATGCCTGGTAAATGGGTTGAAGATAGTGATGAAAGCGGTGAT<br>ACCGATGATCCGGAAGAAGAAGAGGAAGAGGCACCGGCACCGAATGAAGAAGAAACCTGCGAAAAT<br>AATGAGAGCCCGAAAAAAAAAACCTTTAAACGTCGCAAAATCTATTGCCAGATTACCCAGCATCTG<br>CTGCAGAACCATAAAATGTGGAAAAAAGTGATCGAAGAAGAACAGCGTCTGGCAGGTATTGAAAAT<br>CAGAGCCTGGATCAGACACCGCAGAGCCATAGCAGCGAGCAGATTCAGGCCATTAAAGAGGAAGAA<br>GAAGAAAAAGGTAAACCGCGTGGTGAAGAAATTCCGACCCAGAAACCGGATCAG
SLFN12	ATGAACATCTCCGTGGACCTCGAGACTAACTACGCTGAGCTGGTGCTGGACGTGGGTCGTGTGACC<br>CTGGGAGAGAACTCCCGCAAGAAGATGAAGGACTGCAAGCTGCGCAAGAAGCAGAACGAGTCTGTC<br>TCCCGTGCTATGTGCGCTCTGCTGAACTCCGGTGGTGGTGTCATCAAGGCTGAGATCGAGAACGAG<br>GACTACTCCTACACCAAGGACGGTATCGGCCTGGACCTGGAAAACTCCTTCTCCAACATCCTGCTG<br>TTCGTGCCCGAGTACCTGGACTTCATGCAGAACGGCAACTACTTCCTGATCTTCGTGAAGTCCTGG<br>TCCCTGAACACCTCCGGCCTGCGTATCACCACCCTGTCCTCCAACCTGTACAAGCGTGACATCACC<br>TCCGCTAAAGTGATGAACGCTACCGCTGCTCTCGAGTTCCTGAAGGACATGAAGAAGACCCGCGGT<br>CGTCTGTACCTGCGTCCCGAGCTGTTGGCTAAGCGCCCCTGCGTGGACATCCAAGAGGAAAACAAC<br>ATGAAGGCTCTGGCCGGCGTGTTCTTCGACCGTACTGAGCTGGACCGCAAGGAAAAGCTGACCTTC<br>ACCGAGTCCACCCACGTCGAGATCAAGAACTTCTCCACCGAGAAGCTGCTGCAGCGCATCAAGGAA<br>ATCCTGCCCCAGTACGTGTCCGCTTTCGCTAACACCGACGGTGGTTACCTGTTCATCGGCCTCAAC<br>GAGGATAAGGAAATCATCGGTTTCAAGGCCGAGATGTCTGACCTGGACGACCTCGAGCGTGAAATC<br>GAGAAGTCCATCCGCAAGATGCCCGTGCACCACTTCTGCATGGAAAAGAAGAAGATCAACTACTCC<br>TGCAAGTTCCTGGGCGTGTACGACAAGGGTTCCCTGTGCGGTTACGTGTGCGCCCTGCGTGTCGAG<br>AGGTTCTGCTGCGCTGTGTTCGCTAAGGAACCCGACTCCTGGCACGTGAAGGACAACCGTGTGATG<br>CAGCTGACTAGGAAGGAATGGATCCAGTTCATGGTCGAGGCTGAGCCCAAGTTCTCCAGCTCCTAC<br>GAGGAAGTGATCTCCCAGATCAACACCTCCCTGCCCGCTCCCCACTCCTGGCCTTTGTTGGAATGG<br>CAGCGTCAGCGTCACCACTGCCCTGGACTGTCCGGTCGTATCACCTACACCCCCGAGAACCTGTGC<br>CGCAAGCTGTTCCTGCAGCACGAGGGCCTGAAGCAGCTGATCTGCGAAGAGATGGACTCCGTGCGC<br>AAGGGTTCTCTGATCTTCTCTAGGTCTTGGAGCGTGGACCTGGGCCTGCAAGAGAACCACAAGGTG<br>CTGTGCGACGCCCTGCTGATCTCTCAGGACTCCCCCCCTGTGCTGTACACCTTCCACATGGTGCAG<br>GACGAGGAATTCAAGGGCTACTCCACCCAGACCGCTCTGACTCTGAAGCAAAAGCTGGCTAAGATC<br>GGCGGTTACACCAAGAAAGTGTGCGTGATGACCAAGATCTTCTACCTGTCCCCCGAGGGAATGACC<br>TCCTGCCAGTACGACCTGCGTTCCCAAGTGATCTACCCCGAGTCCTACTACTTCACCCGTCGCAAG<br>TACCTGCTGAAGGCCCTGTTCAAGGCTCTGAAGCGTCTGAAGTCCCTGCGTGACCAGTTCAGCTTC<br>GCTGAGAACCTCTACCAGATCATCGGTATCGACTGCTTCCAAAAGAACGATAAGAAGATGTTCAAG<br>TCCTGCCGTCGCCTGACC

Supplementary Table 6. Codon-optimized cDNA sequences for expression of PDE3A and SLFN12.
