# Supplementary material for: Structure of PDE3A-SLFN12 complex reveals requirements for activation of SLFN12 RNase
Source: Nat Commun. 2021 Jul 16;12:4375. doi: 10.1038/s41467-021-24495-w (PMC8285493; doi:10.1038/s41467-021-24495-w)
Supplement: Supplementary file 3 — Description of Additional Supplementary Files [file 41467_2021_24495_MOESM3_ESM.pdf]

Description of Additional Supplementary Files

Supplementary Data 1: HDX data: Deuterium uptake values of $PDE3A^{CAT}$:BRD9500 and $PDE3A^{CAT}$:BRD9500:SLFN12.

Supplementary Data 2: Enrichment scores for mutant alleles in the deep mutational scanning experiment.

Supplementary Movie 1: Dynamics observed between $PDE3A^{CAT}$ and SLFN12 during data collection. Movie depicting the middle 80% of the particle movement range distribution along the most significant (representing ~ 30% of the variance) eigenvector of particle alignment changes between the SLFN12 body and the $PDE3A^{CAT}$ body. Orientation and color scheme is as shown in Figure 5A.
