# Supplementary material for: Structure of PDE3A-SLFN12 complex reveals requirements for activation of SLFN12 RNase
Source: Nat Commun. 2021 Jul 16;12:4375. doi: 10.1038/s41467-021-24495-w (PMC8285493; doi:10.1038/s41467-021-24495-w)
Supplement: Supplementary file 7 — Reporting summary [file 41467_2021_24495_MOESM7_ESM.pdf]

nature research

Corresponding author(s): Heidi Greulich
Last updated by author(s): Jun 9, 2021

Reporting Summary

Nature Research wishes to improve the reproducibility of the work that we publish. This form provides structure for consistency and transparency in reporting. For further information on Nature Research policies, see our Editorial Policies and the Editorial Policy Checklist.

Statistics

For all statistical analyses, confirm that the following items are present in the figure legend, table legend, main text, or Methods section.

n/a	Confirmed	
☐	☒	The exact sample size (n) for each experimental group/condition, given as a discrete number and unit of measurement
☒	☐	A statement on whether measurements were taken from distinct samples or whether the same sample was measured repeatedly
☒	☐	The statistical test(s) used AND whether they are one- or two-sided<br>*Only common tests should be described solely by name; describe more complex techniques in the Methods section.*
☒	☐	A description of all covariates tested
☒	☐	A description of any assumptions or corrections, such as tests of normality and adjustment for multiple comparisons
☐	☒	A full description of the statistical parameters including central tendency (e.g. means) or other basic estimates (e.g. regression coefficient) AND variation (e.g. standard deviation) or associated estimates of uncertainty (e.g. confidence intervals)
☒	☐	For null hypothesis testing, the test statistic (e.g. F, t, r) with confidence intervals, effect sizes, degrees of freedom and P value noted<br>*Give P values as exact values whenever suitable.*
☒	☐	For Bayesian analysis, information on the choice of priors and Markov chain Monte Carlo settings
☒	☐	For hierarchical and complex designs, identification of the appropriate level for tests and full reporting of outcomes
☒	☐	Estimates of effect sizes (e.g. Cohen's d, Pearson's r), indicating how they were calculated

Our web collection on statistics for biologists contains articles on many of the points above.

Software and code

Policy information about availability of computer code

Data collection	No software used other than software that comes with each data collection instrument.
Data analysis	LiCOR Odyssey CLx and Image Studio 2.0 for scanning Western images.<br>Prism 8 for plotting relative CTG viability data and dose-titration curve fitting<br>Benchling.com for DNA sequence alignments<br>CHOPCHOP for sgRNA selection (https://chopchop.cbu.uib.no/)<br>CRISPResso for analyzing CRISPRseq data (https://crispresso.pinellolab.partners.org/submission)<br>Clustal Omega 1.2.4 for multiple sequence alignment (https://www.ebi.ac.uk/Tools/msa/clustalo/)<br>AlphaImager and AlphaView for RNA gel imaging<br>CisTEM 1.0.0beta - Cryo-EM data analysis<br>Relion 3.1 - Cryo-EM data analysis<br>CTFFind4 4.1.13 - Cryo-EM data analysis<br>Phenix 1.18 - Cryo-EM data analysis<br>Chimera 1.13 - Cryo-EM data analysis<br>Coot 0.8.9.2 - Cryo-EM data analysis<br>Spectrum Mill Proteomics Workbench (prerelease version B.06.01.202, Agilent Technologies) for peptide identification<br>HDExaminer (Sierra Analytics) for HDX-MS processing<br>Biophysics and crystallography:<br>XDS v. 20200417<br>PHENIX v. 1.19.1<br>Coot v. 0.9<br>CCP4 v. 7.1

Pymol v. 2.4.0
Astra v. 7.3.2
PRISM v. 7
Fortebio Data Analysis v. 10.0.3.1
MicroCal VP-Capillary DSC Software 2 v. 3.2.10
SedFIT v. 15.01b
SedPHAT v. 12.1b
GUSSI v. 1.4.1
SedNTerp v. 20120828 BETA

For manuscripts utilizing custom algorithms or software that are central to the research but not yet described in published literature, software must be made available to editors and reviewers. We strongly encourage code deposition in a community repository (e.g. GitHub). See the Nature Research guidelines for submitting code & software for further information.

Data

Policy information about availability of data

All manuscripts must include a data availability statement. This statement should provide the following information, where applicable:

- Accession codes, unique identifiers, or web links for publicly available datasets
- A list of figures that have associated raw data
- A description of any restrictions on data availability

PDB accession numbers for the crystal structures in Figure 2 are: PDE3A+cAMP, 7L29; PDE3A+DNMDP, 7KWE; PDE3A+trequinsin, 7L28; PDE3A apo structure, 7L27. The HDX summary data for Figure 2 can be found in Supplementary Dataset 1. The original mass spectra for Figure 2 and the protein sequence database used for searches have been deposited in the public proteomics repository MassIVE (http://massive.ucsd.edu) and are accessible at ftp://MSV000087620@massive.ucsd.edu when providing the dataset password: structure. If requested, also provide the username: MSV000087620. This dataset will be made public upon acceptance of the manuscript. The PDB accession numbers for the Cryo-EM structures in Figure 3 and Figure 4 are: PDE3A, 7LRC; SLFN12, 7LRE, PDE3A-DNMDP-SLFN12 complex, 7LRD. The raw data for the Deep Mutational Scanning experiment in Figure 6 can be found in Supplementary Dataset 2. The EMDB accession numbers for the Cryo-EM structures in Figure 3 and Figure 4 are: PDE3A, EMD-23494; SLFN12, EMD-23496; PDE3A-DNMDP-SLFN12 complex, EMD-23495

Field-specific reporting

Please select the one below that is the best fit for your research. If you are not sure, read the appropriate sections before making your selection.

[x] Life sciences [ ] Behavioural & social sciences [ ] Ecological, evolutionary & environmental sciences

For a reference copy of the document with all sections, see nature.com/documents/nr-reporting-summary-flat.pdf

Life sciences study design

All studies must disclose on these points even when the disclosure is negative.

Sample size	Sample sizes were chosen based on the anticipated variance in the data and the technical limitations of each experiment. A minimum of three replicates were included for any experiment in which standard deviations were calculated.
Data exclusions	No data were excluded.
Replication	For Figure 5B, similar results were obtained using a different ectopic expression system (CMV promoter-driven). For Figure 6C and 6E, similar results were obtained using an independent PDE3A knockout cell line. For Fig 6D, similar complex formation results were obtained using the SLNF12-EEAA variant, which was determined to maintain DNMDP-induced complex formation with PDE3A. For Fig 7A, DNMDP-induced PDE3A-SLFN12 complex formation has been repeated >3 times for wild type and the EEAA mutant of SLFN12. In addition, DNMDP-induced PDE3A-SLFN12 complex formation for wild type and all active site mutants were assayed independently using doxycycline-induced SLFN12 expression in HeLa-Res cells (lacking endogenous SLFN12 expression). Results were very similar for all experimental trials. The E200A and E205A single mutants were only assayed once. For Fig 7B, similar results were obtained using a different ectopic expression system (CMV promoter-driven constitutive expression). All RNase assays in Figure 7 were performed at least twice with consistent results.
Randomization	Cell lines were split into identical samples and randomly allocated for all cell-based experiments.
Blinding	Investigators were not blinded to sample ID in biochemical, biophysical, or cellular experiments in order to track sample identity and avoid errors in sample naming.

Reporting for specific materials, systems and methods

We require information from authors about some types of materials, experimental systems and methods used in many studies. Here, indicate whether each material, system or method listed is relevant to your study. If you are not sure if a list item applies to your research, read the appropriate section before selecting a response.

Materials & experimental systems

n/a	Involved in the study
	☒ Antibodies
	☒ Eukaryotic cell lines
☒	☐ Palaeontology and archaeology
☒	☐ Animals and other organisms
☒	☐ Human research participants
☒	☐ Clinical data
☒	☐ Dual use research of concern

Methods

n/a	Involved in the study
☒	☐ ChIP-seq
☒	☐ Flow cytometry
☒	☐ MRI-based neuroimaging

Antibodies

Antibodies used

anti-PDE3A antibodies (Bethyl A302-740, Bethyl A302-741), anti-FLAG M2 antibody (Sigma-Aldrich F1804), anti-VCL antibody (Sigma-Aldrich V9264), anti-ACTIN antibody (CST 3700), anti-V5 antibody (Thermo Fisher Scientific R960-25), anti-V5 magnetic beads (MBL m167-11)

Validation

Bethyl A302-740: Rabbit anti-PDE3A Antibody, Affinity Purified. Reactivity: Human. Applications: WB, IP. Immunogen: between 450 and 500. (https://www.bethyl.com/product/A302-740A/PDE3A+Antibody).
Bethyl A302-741: Rabbit anti-PDE3A Antibody, Affinity Purified. Reactivity: Human. Applications: WB, IP. Immunogen: between 575 and 625. (https://www.bethyl.com/product/A302-741A/PDE3A+Antibody).

Sigma-Aldrich F1804:The ANTI-FLAG M2 mouse, affinity purified monoclonal antibody binds to fusion proteins containing a FLAG peptide sequence. The antibody recognizes the FLAG peptide sequence at the N-terminus, Met-N-terminus,C-terminus, and internal sites of the fusion protein. For highly sensitive and specific detection of FLAG fusion proteins by immunoblotting, immunoprecipitation (IP), immunohistochemisty, immunofluorescence and immunocyotchemistry. Optimized for single banded detection of FLAG fusion proteins in mammalian, plant, and bacterial expression systems. Western Blotting and EIA. (https://www.sigmaaldrich.com/catalog/product/sigma/f1804?lang=en&region=US).

CST 5174: GAPDH (D16H11) XP® Rabbit mAb detects endogenous levels of total GAPDH protein.(https://www.cellsignal.com/products/primary-antibodies/gapdh-d16h11-xp-rabbit-mab/5174).

Sigma-Aldrich V9264: Monoclonal Anti-Vinculin antibody produced in mouse. Specifically labels vinculin at cell-cell and cell-substrate contacts. Reacts strongly with human vinculin. Shows cross-reactivity with smooth muscle metavinculin.Anti-Vinculin antibody, Mouse monoclonal has been used in
• immunoblotting
• immunofluorescence staining
• immunocytochemistry
• immunohistochemistry and enzyme linked immunosorbent assay (ELISA).
(https://www.sigmaaldrich.com/catalog/product/sigma/v9264?lang=en&region=US)

CST 3700: β-Actin (8H10D10) Mouse mAb detects endogenous levels of total β-actin protein. Due to the high sequence identity between the cytoplasmic actin isoforms, β-actin and cytoplasmic γ-actin, this antibody may cross-react with cytoplasmic γ-actin. It does not cross-react with α-skeletal, α-cardiac, α-vascular smooth, or γ-enteric smooth muscle isoforms. (https://www.cellsignal.com/products/primary-antibodies/b-actin-8h10d10-mouse-mab/3700).

Thermo Fisher Scientific R960-25: R960-25 recognizes amino acid sequence: -Gly-Lys-Pro-Ile-Pro-Asn-Pro-Leu-Leu-Gly-Leu-Asp-Ser-Thr-.

This antibody is functionally tested against 20 ng of an E. coli expressed fusion protein containing a V5 epitope using a chemiluminescent substrate at a 1 minute exposure. This antibody has also been tested in Western blot against 25 ng of recombinant Positope™ protein. The Positope™ control protein is a 53 kDa recombinant protein that contains seven epitope tags, including His (C-term), HisG, c-myc, and V5. Low background was observed using chemiluminescent or alkaline phosphatase reagents for detection. For Western blot, dilute in PBS or Tris-Buffered Saline (TBS) containing 0.05% Tween-20 and 5% nonfat, dry milk (PBSTM or TBSTM).

Using chemiluminescence as the detection method, no cross-reactivity has been observed in bacterial lysates. In mammalian lysates, a few cross-reactive proteins have been observed upon overexposure of blots. (https://www.thermofisher.com/antibody/product/V5-Tag-Antibody-Monoclonal/R960-25).

MBL m167-11: Monoclonal Antibody conjugated to magnetic beads of 20 tests targeting V5-tag for IP. (https://www.mblintl.com/products/m167-11/)

Eukaryotic cell lines

Policy information about cell lines

Cell line source(s)	HeLa - ATCC (CCL-2)<br>HEK293T - ATCC (CRL-3216)<br>A2058, GB1 - Cancer Cell Line Encyclopedia<br>SF9 - Expression Systems
Authentication	HeLa and A2058 cells were obtained directly from ATCC. GB1 cells were obtained from Cancer Cell Line Encyclopedia. No additional authentication experiments were performed.
Mycoplasma contamination	The cell lines were not further tested for mycoplasma contamination.
Commonly misidentified lines<br>(See ICLAC register)	None.
